# Longitudinal Associations Between Core Self-Evaluation, Vital Exhaustion and Hair Cortisol in Teachers and the Mediating Effects of Resignation Tendency

**DOI:** 10.3389/fpsyg.2022.907056

**Published:** 2022-07-07

**Authors:** Sandra Schneider, Alexander Wettstein, Wolfgang Tschacher, Loredana Torchetti, Gabriel Jenni, Fabienne Kühne, Martin grosse Holtforth, Roberto La Marca

**Affiliations:** ^1^Department of Research and Development, University of Teacher Education Bern, Bern, Switzerland; ^2^Clinical Psychology and Psychotherapy, Department of Psychology, University of Bern, Bern, Switzerland; ^3^Experimental Psychology Division, University Hospital of Psychiatry and Psychotherapy, University of Bern, Bern, Switzerland; ^4^Psychosomatic Medicine, Department of Neurology, Inselspital, Bern University Hospital, University of Bern, Bern, Switzerland; ^5^Clinica Holistica Engiadina, Centre for Stress-Related Disorders, Susch, Switzerland; ^6^Clinical Psychology and Psychotherapy, Department of Psychology, University of Zurich, Zürich, Switzerland

**Keywords:** core self-evaluation, teacher stress, vital exhaustion, hair cortisol, resignation tendency, longitudinal design

## Abstract

Work-related stress appears to be especially high among teachers. However, most research on teacher stress relies exclusively on teachers’ self-reports. Little is known about the physiological correlates of affective stress in teachers. This longitudinal study investigates the relationship between core self-evaluation and adverse psychological and physiological stress outcomes in 42 teachers. Self-report questionnaires were used to assess core self-evaluation, vital exhaustion, and resignation tendency. The concentration of cortisol was assessed using hair samples. One year after the initial measurement, vital exhaustion and hair cortisol were assessed again. Path-analytic mediational models showed that core self-evaluation strongly predicted vital exhaustion, and resignation tendency partially mediated this relationship. However, core self-evaluation did not predict hair cortisol concentration. These findings suggest that core self-evaluation plays a crucial role in preventing vital exhaustion among teachers. A positive core self-evaluation seems beneficial for teachers’ primary and secondary appraisal and an essential resource for the long-term prevention of self-reported vital exhaustion.

## Introduction

Teachers are considered a particularly stressed occupational group (Kyriacou, 2015) with above-average burnout rates compared to other professions (de Heus and Diekstra, 1999; Aloe et al., 2014). Approximately 30% of teachers report that the teaching profession is “very stressful” or “extremely stressful” (Kunz Heim et al., 2014). Teachers’ work stress has been found to contribute to personal suffering, high economic costs (Künzi and Oesch, 2016), and reduced teaching quality (Klusmann et al., 2016). Consequently, many teachers leave the job or retire early (Ingersoll, 2001; Weber et al., 2003; Friedman, 2006). Considering these far-reaching consequences, research is needed to identify protective factors preventing chronic stress in teachers. The personality construct “core self-evaluation” may play a key role in preventing teacher stress, as previous studies have shown positive associations between core self-evaluation and life—and job satisfaction (Judge et al., 1997). Furthermore, some studies suggest that core self-evaluation may be a personal resource lowering the risk of burnout (Best et al., 2005; Brunborg, 2008; Alarcon et al., 2009; Greaves et al., 2017). The present study aims to improve our understanding of core self-evaluation as a potential protective factor of adverse stress-related health outcomes measured *via* teachers’ vital exhaustion and hair cortisol concentrations (HCC).

### Core Self-Evaluation—A Protective Factor for Teacher Stress?

*Core self-evaluation* is a higher-order construct subsuming four well-known traits: global self-esteem, generalized self-efficacy, emotional stability (low neuroticism), and internal locus of control (Judge et al., 1997). Judge et al. (1998) assume that core self-evaluation represents a relatively stable personality trait encompassing fundamental beliefs about oneself and one’s functioning in the environment. The subconstruct self-esteem refers to the individuals’ appraisal of their self-worth and the overall value they attribute to themselves. Generalized self-efficacy represents the appraisal of how well one can cope with life’s challenges in various situations (Bandura, 1977). Emotional stability reflects the tendency to feel calm and balanced and be less reactive to everyday events. In contrast, individuals with high levels of emotional lability, i.e., neuroticism, are prone to experience negative feelings such as anxiety, show poor emotional adjustment, and react more strongly to daily hassles (McCrae and Costa, 1986). Internal locus of control means believing in one’s ability to influence the environment and achieve desired effects in a way that one’s environment and outcomes are controllable (Rotter, 1966).

Based on a transactional model of stress (Lazarus and Folkman, 1984; Friedman-Krauss et al., 2014; McCarthy et al., 2016), core self-evaluation can be understood as a resource that may affect teachers’ primary and secondary appraisal (Judge et al., 1997). During the *primary appraisal*, which is the evaluation of the situation itself, core self-evaluation can be interpreted as the lens through which teachers view their classroom environment, impacting their appraisal of external events and experiences. During the *secondary appraisal*, core self-evaluation may contribute to successful self-regulatory functioning by positively influencing teachers’ evaluation of coping styles and actions in challenging teaching situations.

*In sum*, a positive core self-evaluation can be interpreted as “fundamental premises that individuals hold about themselves and their functioning in the world” (Judge et al., 1998, p. 168) and may positively affect teachers’ perceptions, emotions, cognitions, and actions in challenging classroom situations.

### Resignation Tendency

Coping plays a crucial role in mediating the impact of stress on the individual (Serwinski, 2017). Depending on the perceived resources, coping styles range from active, problem-focused to passive, avoidant, emotion-focused coping styles (Finset et al., 2002). Resignation tendency is a passive, avoidant, emotion-focused coping style and is used when individuals believe to have no control and cannot make a difference (Finset et al., 2002). Consequently, they quickly resign and give up (Schaarschmidt and Fischer, 2008). Avoidant coping styles such as passive resignation may reduce stress resilience in the long term as the underlying problem causing the stress response remains unresolved. They may deplete resources and contribute to teachers’ stress experience and emotional exhaustion in the long run (Ito and Brotheridge, 2003). In contrast, functional coping styles such as active problem solving are negatively related to emotional exhaustion (De Rijk et al., 1998).

Studies examining the relationship between personality and coping styles show that positive core self-evaluation is more associated with active coping styles (Best et al., 2005; Kammeyer-Mueller et al., 2009), whereas neuroticism is more related to passive, avoidant coping styles (Connor-Smith and Flachsbart, 2007). Avoidant coping styles have been associated with vital exhaustion and elevated hair cortisol concentration. van Zijderveld et al. (2013) found that an avoidant, emotion-focused coping style was positively related to vital exhaustion. Serwinski (2017) found that avoidant coping approaches are associated with elevated hair cortisol concentrations under low-but, not high-stress conditions.

### Psychophysiological Stress Consequences

The appraisal of a situation as potentially threatening results in psychological, physiological, and behavioral stress responses (Lazarus, 1991; Mauss and Robinson, 2009). Research in educational psychology on teacher stress has primarily focused on the psychosocial stress experience of teachers, neglecting physiological stress reactions. Physiologically, *acute stress* prepares the body for a challenge and is not harmful for the time being (Birbaumer and Schmidt, 2010). *Chronic stress*, however, endangers teachers’ health and wellbeing by potentially leading to long term physiological (e.g., altered autonomous nervous system activity, hypothalamic-pituitary-adrenal axis dysfunctions, subclinical inflammation), psychosomatic (e.g., sleep disorders, depressiveness, anxiousness) (Bellingrath et al., 2008; Qi et al., 2014) and social (e.g., withdrawal, social insecurity) stress consequences (Brouwers and Tomic, 2000; Kokkinos, 2007; Klusmann et al., 2016).

Vital exhaustion is a stress-induced outcome on a psychosomatic level. Vital exhaustion refers to a state of unusual fatigue, lack of energy, irritability, and feelings of demoralization and is considered a potential early warning sign of cardiovascular disease (Appels and Mulder, 1989). Whereas the process of vital exhaustion is not yet fully understood, it may result from a failed adaptation to chronic stress (Schoch et al., 2018) and is closely related to the concept of burnout. Women generally report higher levels of vital exhaustion than men, which may be due to psychological and physiological sex differences in the stress response (Prescott et al., 2003; Verma et al., 2011; Frestad and Prescott, 2017).

Hair cortisol concentration (HCC) is a stress-induced outcome on a physiological level. The detection of HCC is a relatively new strategy to measure long-term cumulative cortisol levels (Russell et al., 2012). Systemic cortisol exposure during specific periods can be analyzed by segmenting a hair sample. Previous studies on the relationship between self-reported stress and HCC have been inconsistent. In about half of the studies, no significant associations were found (Kudielka et al., 2006; Gidlow et al., 2016; Noser et al., 2018; Rohleder, 2018).

### Mediating Effects of Resignation Tendency

Beyond the described direct effects of core self-evaluation and resignation tendency on teachers’ stress outcomes, it can be assumed that a positive core self-evaluation prevents vital exhaustion in teachers also indirectly *via* a reduced resignation tendency. Thus, a positive core self-evaluation may not only lead to a more positive primary appraisal of the environment but also directly affect teachers’ coping styles. During the secondary appraisal, core self-evaluation may contribute to successful self-regulatory functioning by positively influencing teachers’ evaluation of coping styles and actions in challenging teaching situations. Hence, we expect that core self-evaluation is indirectly associated with teachers’ vital exhaustion *via* a reduced resignation tendency. Considering not only direct but also indirect associations might further deepen the understanding of the interplay between core self-evaluation, resignation tendency, and teachers’ psychophysiological stress outcomes.

### The Present Study

Previous empirical studies on teacher stress have primarily relied on self-reports and have focused mainly on the subconstructs of emotional stability or self-efficacy (Schwarzer and Hallum, 2008; Schwerdtfeger et al., 2008; Zee and Koomen, 2016) rather than the full range of constructs encompassed by core self-evaluation. The present study extends this limited approach and explores the potential of the whole construct of core self-evaluation in predicting and protecting against psychosomatic and physiological stress responses in teachers in a cross-sectional and longitudinal design.

First, we focus on associations between core self-evaluation, vital exhaustion, and HCC in cross-sectional (t1) data, complemented by the prediction of vital exhaustion and HCC by core self-evaluation in longitudinal data 1 year later (t2), controlling for the initial measurement. Based on previous research on the relationship between core self-evaluation, stress appraisal, and burnout (Best et al., 2005; Brunborg, 2008), we expect a significant negative association between core self-evaluation and vital exhaustion in teachers at t1 and a weaker but still significant association between these constructs 1 year later at t2 (Hypothesis 1). Second, we explore the association between teachers’ core self-evaluation and hair cortisol concentrations HCC (Hypothesis 2). We are not aware of any studies on HCC and core self-evaluation, making accurate predictions challenging. However, a study by Erickson et al. (2021) found a positive association between the core self-evaluation subconstruct neuroticism, i.e., low emotional stability, and HCC. Furthermore, studies found negative associations between core self-evaluation and self-reported job stress (Brunborg, 2008) and job burnout (Best et al., 2005). Given that self-reported and physiological stress seem to be only partially associated (Gidlow et al., 2016), we expect only weak negative associations between core self-evaluation and HCC levels at t1 and t2. Third, we examine if the dysfunctional coping style resignation tendency mediates the relationship between core self-evaluation and vital exhaustion (Hypothesis 3). Based on the transactional stress model and prior studies on coping styles and personality (Connor-Smith and Flachsbart, 2007; Kammeyer-Mueller et al., 2009), and avoidant coping styles and vital exhaustion (van Zijderveld et al., 2013), we expect that resignation tendency negatively mediates the relationship between core self-evaluation and vital exhaustion at least partially (Serwinski, 2017). Fourth, we investigate if resignation tendency negatively mediates the relationship between core-self-evaluation and HCC (Hypothesis 4).

## Materials and Methods

### Participants

We conducted an *a priori* Monte Carlo power analysis for the mediating effects of core self-evaluations over resignation tendency on vital exhaustion with the online calculator by Schoemann and colleagues (Schoemann et al., 2017). The analysis showed that with power = 0.80, alpha level = 0.05 and large expected effect sizes (*r* = 0.50 based on past research) the required sample size is *N* = 70. Past research is more ambiguous for the analyses involving HCC, so the power calculation focused on the direct effect of core self-evaluation on HCC. We expected a moderate association (*r* = 0.35), resulting in a required sample size of *N* = 49, calculated with the software G*Power (Faul et al., 2009).

Initially, we received requests from 76 teachers, of which 21 were excluded due to the following criteria: chronic diseases (*n* = 8), medication (*n* = 5), contact termination (*n* = 4), living and working outside canton [outside the canton of Bern, Switzerland] (*n* = 1), teaching less than 16 lessons per week (*n* = 1), pregnancy (*n* = 1), and traveling outside [the canton of Bern, Switzerland] during data collection (*n* = 1). Before the first data collection, *n* = 7 teachers withdrew their participation for personal reasons not further specified (*n* = 4), being too stressed (*n* = 2), or a lack of interest (*n* = 1). Forty-eight teachers completed the first online questionnaire, of which *n* = 6 dropped out due to stress (*n* = 3), medication (*n* = 1), job change (*n* = 1), and personal reasons not further specified (*n* = 1). This final sample size of 42 healthy teachers (28 females, *M*_*age*_ = 39.66, *SD* = 11.99) completed the first online questionnaire and attended the first measurement of physiological variables (t1) at the beginning of 2020 before the COVID-19 pandemic. On average, the final sample revealed 13.35 years of teaching experience (*SD* = 11.07, range = 1–40). They completed teacher training and were regular classroom teachers, not gifted or special education teachers. The grade levels they taught ranged from kindergarten and elementary school (kindergarten to 6th grade; *n* = 27) to secondary school (7th to 9th grade; *n* = 12) and to high school and vocational school (10th to 12th grade; *n* = 3). Three participants were either bald or had too short hair to take a hair sample to determine the cortisol concentration, resulting in 39 teachers providing self-reports as well as physiological data at the first measurement. When completing the follow-up measurement 1 year later (t2), during the COVID-19 pandemic, three more teachers withdrew their participation due to moving abroad (*n* = 1) and pregnancy (*n* = 2), resulting in 39 teachers with follow-up self-report data and 36 teachers with follow-up self-report data as well as follow-up physiological data, respectively.

All teachers were screened in a short interview to gather information on demographic variables (sex, age, years of teaching experience, number of lessons taught per week) and to ensure that inclusion criteria were met. The participating teachers signed informed consent. The study was conducted in strict compliance with current data protection laws and was approved by the ethics committee of [the canton of Bern], and the Internal Review Board (IRB) of the University of [Bern].

### Procedures

The present study is part of a larger, longitudinal study examining psychobiological stress in teachers. Participants completed online questionnaires on vital exhaustion and resignation tendency before the COVID-19 pandemic in December 2019. Hair samples were collected at the University of [Bern] before the pandemic between January and February 2020. During the COVID-19 pandemic in 2020, teachers were teaching in person, not digitally. One year after the initial measurement (t2), participants completed another online questionnaire on vital exhaustion and provided another hair sample.

### Measures

#### Self-Reports

*Core Self-Evaluation* was assessed using a validated German version (Heilmann and Jonas, 2010) of the original core self-evaluation scale by Judge et al. (2003). Sample Items are: *“I am confident of getting the success I deserve in life,” “When I try hard, I am generally successful.”* Items were rated on a five-point Likert scale from “absolutely disagree” (1) to “absolutely agree” (5). Mean values were calculated. The reliability of this twelve-item scale was α = 0.78.

*Resignation tendency* was assessed with the subscale with the same name of the Measure of Coping Capacity Questionnaire (MECCA; Schaarschmidt and Fischer, 2008). Participants rated each of the six items with a value between “absolutely disagree” (1) to “absolutely agree” (5). Sample items are *“If I fail, I resign quickly,” “I easily lose heart when I don*’*t succeed despite the effort.”* This resulted in a total sum score ranging from 6 to 30 points. The reliability was α = 0.84.

*Vital exhaustion* was assessed at t1 and t2 (1-year follow-up) with the German translation (cf. Rau et al., 2010) of the Maastricht Vital Exhaustion Questionnaire (MQ; Appels et al., 1987). The scale consists of 21 items assessing fatigue, difficulties falling asleep, general malaise, apathy, irritability, energy loss, depression, and waking up exhausted. Sample items were: *“Do you sometimes feel as if your body is like a battery that is losing its power?,” “Do you have increasing difficulty in concentrating on a single subject for long?”* The 21 items can be answered with “Yes,” “No,” or “?.” Each item could be rated on a three-point scale, from “statement is not true” (1) to “undecided” (2) or “true” (3). The scores of each item can be added together to calculate the total score, with higher scores indicating increased vital exhaustion. The reliability was α = 0.88 at t1 and α = 0.88 at t2.

#### Hair Cortisol Concentration

Hair strands were cut from the posterior vertex as close to the scalp as possible. Hair cortisol concentrations were determined from the 3-cm segment closest to the scalp. Given an average hair growth of 1 cm per month (Wennig, 2000), this segment represents the cumulative glucocorticoid secretion over 3 months before sampling. The washing procedure and glucocorticoid extraction followed the laboratory protocol described by Gao et al. (2013). All samples were analyzed by liquid chromatography coupled with tandem mass spectrometry (LC-MS/MS). This analysis’s lower limits of quantification (LOQ) were below 0.1 pg/mg for cortisol. The inter-and intra-assay coefficients of variance for cortisol were below 15% (Gao et al., 2013).

### Data Analyses

The Shapiro-Wilk test was used for each variable to test whether the data were normally distributed. The variables vital exhaustion and HCC were log-transformed due to non-normal distribution. Descriptive statistics and bivariate correlations were computed to investigate the study’s variables. The research questions were analyzed using path-analytic mediation models (Hayes, 2018). First, we investigated the total effects (effect *c*) of teachers’ core self-evaluation on vital exhaustion and HCC (H1 and H2, respectively) cross-sectionally at t1 and t2, controlling for the initial measurement. Then, mediations were examined through the indirect effect (effect *ab*), a multiplicative term built with the coefficients of the effect of teachers’ core self-evaluation on resignation tendency (effect *a*) and the effect of resignation tendency on vital exhaustion and HCC (effect *b*; H3 and H4, respectively). Again, both the cross-sectional and the longitudinal effects were analyzed.

The mediations were tested using ordinary least square path analytic models in SPSS 28 with the macro PROCESS version 4.0 (Hayes, 2018). The significance of the indirect effects was tested with a 95% confidence interval based on 5,000 bias-corrected bootstrap samples. Participants’ sex and age were controlled for in the models. The assumptions of multiple linear regression were tested and indicated some heteroscedasticity in the models predicting vital exhaustion at t2. Therefore, the heteroscedasticity-consistent estimator HC-3 implemented in PROCESS was applied for those models.

## Results

### Associations Between Demographics, Core Self-Evaluation, Resignation Tendency, Vital Exhaustion, and Hair Cortisol Concentrations

Means, standard deviations, and bivariate correlations between core self-evaluation, resignation tendency, vital exhaustion, and HCC are shown in Table 1. All correlations were in the expected direction. Higher levels of core self-evaluation were negatively correlated with resignation tendency and vital exhaustion at t1 and t2. Higher levels of resignation tendency were positively correlated with vital exhaustion at t1 and t2. Vital exhaustion and HCC were neither associated at t1 nor at t2. Vital exhaustion and hair cortisol showed a considerable longitudinal stability over the course of a year, and while levels of vital exhaustion did not differ between t1 and t2 [repeated measures *t*-test: *t*(38) = −0.1.33, *p* = 0.190], HCC decreased significantly [*t*(36) = 2.197, *p* < 0.05]. Finally, male teachers in our sample were older than female teachers and reported less vital exhaustion at t2. Older teachers reported a lower resignation tendency than younger teachers.

**TABLE 1:** Descriptive statistics and intercorrelations of key variables.

Variable	*M*	*SD*	1.	2.	3.	4.	5.	6.	7.
1. Sex*^a^*	−	−	−						
2. Age*^years^*	39.66	11.99	0.35*	−					
3. Core self-evaluation	4.00	0.47	0.05	0.11	−				
4. Resignation tendency	15.19	4.50	−0.27*^t^*	−0.33*	−0.56***	−			
5. Vital exhaustion t1 (log)	31.48	8.71	–0.18	–0.15	−0.75***	0.58***	−		
6. HCC pg/mg t1 (log)	7.8	7.6	0.17	0.07	–0.11	–0.12	0.10	−	
7. Vital exhaustion t2 (log)	32.23	7.95	−0.37*	–0.24	−0.54***	0.46**	0.78***	0.03	−
8. HCC pg/mg t2 (log)	5.8	4.2	0.15	–0.08	0.04	–0.27	–0.06	0.52***	–0.19

*N = 42 (28 females), HCC and vital exhaustion t1: N = 39, HCC t2: N = 36.*

*^a^Sex: 0, female; 1, male.*

*M and SD of vital exhaustion and HCC are reported in raw metrics, while for the correlations these variables were log-transformed.*

^t^p < 0.10, *p < 0.05, **p < 0.01, ***p < 0.001, two-tailed.

### Association Between Core Self-Evaluation and Vital Exhaustion

To test the association between core self-evaluation and vital exhaustion (H1), the total effect *c* (i.e., without the mediator) was examined. At t1, the standardized coefficient was *c_*t*1_* = −0.73, *p* < 0.001, explaining a large amount of variance [*F*_(3_, _38)_ = 16.33, *p* < 0.001, *R^2^_*model*_ _*with controlvariables*_* = 0.56, *R^2^_*core self*–*evaluation*_* = 0.52]. Core self-evaluation t1 was not associated with vital exhaustion at t2 (*c_*t*2_* = 0.02, *p* = 0.887, *R^2^_*core self*–*evaluation*_* = 0.00).

### Association Between Core Self-Evaluation on Hair Cortisol Concentrations

The total effect *c* of core self-evaluation on HCC (H2) was not significant at t1, *c_*t*1_* = −0.14, *p* = 0.412 [*F*_(3_, _35)_ = 0.59, *R^2^_*model*_* = 0.05, *R^2^_*core self*–*evaluation*_* = 0.02]. Longitudinally, core self-evaluation was not associated with HCC at t2, *c_*t*2_* = 0.11, *p* = 0.475, *R^2^_*core self*–*evaluation*_* = 0.01.

### Mediating Effects of Resignation Tendency

#### Indirect Effect of Core Self-Evaluation on Vital Exhaustion *via* Resignation Tendency

**FIGURE 1:**
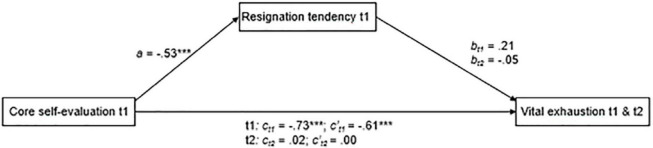
Results of the path-analytical model testing indirect effects of core self-evaluation over resignation tendency on vital exhaustion t1 (*N* = 42) and t2, accounting for vital exhaustion t1 (*N* = 39). To simplify the presentation, we standardized the coefficients and omitted error terms and effects of control variables.

The results of the mediation analyses are shown in Figure 1. There was a significant negative effect of core self-evaluation on resignation tendency, *a* = −0.53, *p* < 0.001 [*F*_(3_, _38)_ = 8.75, *R^2^_*model*_* = 0.41], but no significant effect of resignation tendency on vital exhaustion t1 (*b_*t*1_* = 0.21, *p* = 0.132). However, this resulted in a significant indirect effect (a*b_*t*1_ = −0.11, 95% CI: -0.2825 to -0.0002), indicating that a positive core self-evaluation was associated with lower vital exhaustion by means of a lower resignation tendency. After accounting for the indirect effect, core self-evaluation still had a direct negative effect on vital exhaustion, *c’_*t*1_* = −0.61, *p* < 0.001.

In the longitudinal analysis, the effect of resignation tendency on vital exhaustion t2 was also not significant (*b_*t*2_* = −0.05, *p* = 0.721), indicating no significant indirect effect (a*b_*t*2_ = 0.03, 95% CI: −0.1188 to 0.1805). In contrast to the cross-sectional analysis, there was no direct effect of core self-evaluation on vital exhaustion t2, when the indirect effect and vital exhaustion t1 were controlled (*c’_*t*2_* = 0.00, *p* = 0.983) (**H3**).

**FIGURE 2:**
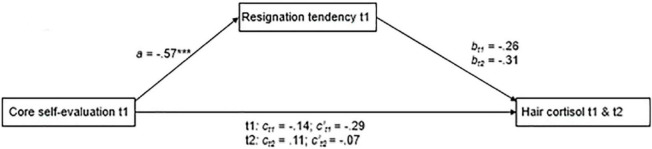
Results of the path-analytical model testing indirect effects of core self-evaluations over resignation tendency on HCC t1 (*N* = 39) and t2, accounting for HCC t1 (*N* = 36). To simplify the presentation, we standardized the coefficients and omitted error terms and effects of control variables.

*Indirect effect of core self-evaluation over resignation tendency on HCC:* Figure 2 shows the results of the mediation analysis including HCC. The negative effect of core self-evaluation on resignation tendency was significant, *a* = −0.57, *p* < 0.001, [*F*_(3, 35)_ = 8.99, *R^2^_*model*_* = 0.44]. The negative effect of resignation tendency on HCC at t1 was not significant (*b_*t*1_* = −0.26, *p* = 0.245), and neither was the corresponding indirect effect (a*b_*t*1_ = 0.15, 95% CI: -0.1021 to 0.4471) After accounting for the indirect effect, core self-evaluation was not significantly associated with HCC at t1 (*c’_*t*1_* = −0.29, *p* = 0.178).

In the longitudinal analysis, neither the effect of resignation tendency on HCC t2 (*b_*t*2_* = −0.31, *p* = 0.133) nor the indirect effect (a*b_*t*2_ = 0.18, 95% CI: -0.0323 to 0.4701) reached significance. As in the cross-sectional analysis, the direct effect of core self-evaluation on HCC t2 was not significant when the indirect effect and HCC t1 were accounted for (*c’_*t*2_* = −0.07, *p* = 0.710) (**H4**).

## Discussion

The primary aim of the current study was to investigate the potential of core self-evaluation in preventing vital exhaustion and elevated hair cortisol concentrations (HCC) in 42 teachers using a combined cross-sectional and longitudinal design. Furthermore, we examined whether this relationship was mediated by resignation tendency.

Compared to the respective norm samples (Heilmann and Jonas, 2010), the examined teachers showed a slightly above-average core self-evaluation, an average resignation tendency (Schaarschmidt and Fischer, 2008), and mild to moderate vital exhaustion (Appels et al., 1987). Overall, the absence of significant deviations from the norm indicates that our sample consisted of primarily healthy teachers without pronounced clinical symptoms.

Our first hypothesis was partially supported as core self-evaluation and vital exhaustion were significantly associated at t1 and t2. Core self-evaluation strongly predicted vital exhaustion at t1 and thereby qualified as a key variable in preventing vital exhaustion with an explained variance of 52%. This result aligns with findings in related fields (Brunborg, 2008; Chang et al., 2012) that identified negative associations within the range of *r* = 0.40 between core self-evaluation and occupational stress. A positive core self-evaluation seems to influence the primary appraisal of the environment positively. In this sense, one might say, “Tell me how you see the world, and I’ll tell you how stressed you feel.” However, core self-evaluation also seems to influence secondary appraisal and thus coping styles such as resignation tendencies. Accordingly, one might say, “Tell me how you appraise your abilities, and I’ll tell you how you’ll cope with the problem.” In this way, the influence of core self-evaluation may go beyond a self-serving biased perception and potentially also influences the teacher’s actions in the classroom and thus the teaching quality.

Contrary to our expectation, core self-evaluation did not predict increased vital exhaustion t2 when controlling for vital exhaustion t1. Vital exhaustion showed a very high longitudinal stability of *r* = 0.78 over 1 year between t1 and t2. Thus, not much variance remains that other variables could explain. Cross-sectional analysis at t2 without controlling for t1 shows similar effects to those at t1. Furthermore, our results show sex differences in vital exhaustion at t2. These results may be explained by women’s mental health being affected more strongly by COVID-19-related measures than men’s, as suggested in the study by Adams-Prassl et al. (2020).

Hypothesis 2, that core self-evaluation and HCC are weakly associated at t1 and t2, was supported. Core self-evaluation does not predict HCC. Neither core self-evaluation nor resignation tendency significantly contributed to the explained variance of HCC. Moreover, vital exhaustion and HCC showed almost zero correlations. Previous studies have also reported a disassociation between the experience of self-reported stress and HCC (Pennebaker, 1982; Mauss and Robinson, 2009; Campbell and Ehlert, 2012). Lacking correspondence between self-reported stress levels and HCC may also be due to methodological differences or confounders (Kudielka et al., 2006). Empirically, Noser et al. (2018) found no associations between vital exhaustion and HCC. A review on self-reported stress and HCC revealed significant results in less than half of the studies (Gidlow et al., 2016), whereas a meta-analysis conducted by Stalder et al. (2017) showed no association. There are various possible explanations for this disassociation. First, vital exhaustion in our non-clinical sample of healthy teachers may not have reached a certain threshold to detect an association with HCC (van der Meij et al., 2018). Second, following Rohleder (2018), there may be individuals with high HCC and others with decreased HCC within a larger sample, depending on how long chronic stress has been experienced. Consequently, no significant relationship to self-reports can be found. Finally, the disassociation between self-reported stress and HCC may be due to difficulties in perceiving one’s (physiological) state adequately (Pennebaker, 1982; Mauss and Robinson, 2009; Campbell and Ehlert, 2012; Wettstein, 2012; Gidlow et al., 2016; Stalder et al., 2017). The latter could lead to teachers suffering from unfavorable physiological stress consequences endangering their health in the long run without realizing it. It is, therefore, essential to take physiological variables into account that may help reveal potentially unnoticed risk factors at an early stage.

Hypothesis 3, that resignation tendency mediates the relationship between core self-evaluation and vital exhaustion, was supported. Mediation analyses revealed that resignation tendency partially mediates the relationship between core self-evaluation and vital exhaustion. The mediating effect of resignation tendency suggests that core self-evaluation positively affects the primary appraisal, i.e., situational appraisal, and partially the secondary appraisal, i.e., the evaluation of coping styles. This aligns with the findings of a study by van Zijderveld et al. (2013) that found a positive relationship between an avoidant, emotion-focused coping style and vital exhaustion, as well as other findings on the interplay between personality and coping styles (Connor-Smith and Flachsbart, 2007).

Hypothesis 4, that resignation tendency mediates the relationship between core self-evaluation and HCC, was not supported. We found only a negative effect of core self-evaluation on resignation tendency at t1. Neither the effect of resignation tendency on HCC nor the indirect effect reached significance at t1 and t2.

### Practical Implications

The findings suggest that core self-evaluation should be given more attention in research on teacher stress and teacher training. For researchers, the construct of core self-evaluation holds great potential for understanding teachers’ stress experience and its interaction with coping styles and adverse health outcomes. In teacher training, prospective teachers should become familiar with core self-evaluation and functional coping styles early on to better understand how they can be strengthened and applied in challenging classroom situations. Core self-evaluation is a relatively stable personality trait, and improvement is difficult (Styvaert, 2011). Still, addressing unrealistically high self-expectations, educating teachers to compensate for negative core self-evaluation with other personal and social resources, and promoting functional coping styles may be beneficial for stress management in the teaching profession and to counteract adverse health outcomes.

### Limitations and Strengths

This study has some limitations. First, the findings are based on a small sample of healthy and medication-free teachers and cannot be generalized to the entire population. Second, we only investigated the effect of core self-evaluation on vital exhaustion, and it is conceivable that vital exhaustion also influences core self-evaluation. Unfortunately, we cannot examine possible reciprocal, transactional relationships between these two constructs longitudinally as we measured core self-evaluation only at the initial assessment. Future research could examine the role of core self-evaluation in a broader range of active and passive coping styles. In addition, future longitudinal studies in clinical samples could deepen our understanding of the emergence and interaction between individual stress appraisal through core self-evaluation, coping styles, and health outcomes.

The present study also has significant strengths. First, it explores the potential of core self-evaluation in predicting vital exhaustion and hair cortisol concentrations in teachers in a combined cross-sectional and longitudinal design. Second, research on teacher stress is usually limited to self-reports. This study combines self-report and physiological data, improving our understanding of the interplay between psychological and physiological processes.

## Conclusion

Core self-evaluation seems to be a crucial resource in preventing vital exhaustion among teachers. A positive core self-evaluation benefits teachers’ primary and secondary appraisal, affecting directly and indirectly vital exhaustion mediated by resignation tendency. Thus, a positive core self-evaluation is an essential resource for the long-term prevention of self-reported vital exhaustion. In contrast, a negative core self-evaluation seems to unfold a maladaptive effect. It is a risk factor for vital exhaustion directly and indirectly over a high resignation tendency in the short term. The present findings suggest that core self-evaluation plays an essential role in influencing teacher health and may be a promising variable for research on teacher stress prevention.

## Data Availability Statement

The raw data supporting the conclusions of this article will be made available by the authors, without undue reservation.

## Ethics Statement

The studies involving human participants were reviewed and approved by the Ethics Committee of the Canton Bern and the Internal Review Board (IRB) of the University of Bern. The patients/participants provided their written informed consent to participate in this study.

## Author Contributions

AW and RLM designed the research. SS and FK performed the assessments. LT, GJ, and AW analyzed the data. AW prepared the first draft. WT, SS, FK, RLM, and MgH provided insightful comments that critically improved the manuscript quality. All authors contributed to the article and approved the submitted version.

## Conflict of Interest

The authors declare that the research was conducted in the absence of any commercial or financial relationships that could be construed as a potential conflict of interest.

## Publisher’s Note

All claims expressed in this article are solely those of the authors and do not necessarily represent those of their affiliated organizations, or those of the publisher, the editors and the reviewers. Any product that may be evaluated in this article, or claim that may be made by its manufacturer, is not guaranteed or endorsed by the publisher.
